# A Moonlighting Human Protein Is Involved in Mitochondrial Import of tRNA

**DOI:** 10.3390/ijms16059354

**Published:** 2015-04-24

**Authors:** Maria Baleva, Ali Gowher, Piotr Kamenski, Ivan Tarassov, Nina Entelis, Benoît Masquida

**Affiliations:** 1 Department of Molecular and Cellular Genetics, UMR 7156 Génétique Moléculaire, Génomique, Microbiologie (GMGM), CNRS—Université de Strasbourg, 67084 Strasbourg, France; E-Mails: mary-bw@mail.ru (M.B.); qureishi83@gmail.com (A.G.); i.tarassov@unistra.fr (I.T.); n.entelis@unistra.fr (N.E.); 2 Department of Molecular Biology, Biology Faculty of Moscow State University, 119992 Moscow, Russia; E-Mail: piotr.kamenski@gmail.com

**Keywords:** human mitochondria, tRNA targeting, enolase

## Abstract

In yeast *Saccharomyces cerevisiae*, ~3% of the lysine transfer RNA acceptor 1 (tRK1) pool is imported into mitochondria while the second isoacceptor, tRK2, fully remains in the cytosol. The mitochondrial function of tRK1 is suggested to boost mitochondrial translation under stress conditions. Strikingly, yeast tRK1 can also be imported into human mitochondria *in vivo*, and can thus be potentially used as a vector to address RNAs with therapeutic anti-replicative capacity into mitochondria of sick cells. Better understanding of the targeting mechanism in yeast and human is thus critical. Mitochondrial import of tRK1 in yeast proceeds first through a drastic conformational rearrangement of tRK1 induced by enolase 2, which carries this freight to the mitochondrial pre-lysyl-tRNA synthetase (preMSK). The latter may cross the mitochondrial membranes to reach the matrix where imported tRK1 could be used by the mitochondrial translation apparatus. This work focuses on the characterization of the complex that tRK1 forms with human enolases and their role on the interaction between tRK1 and human pre-lysyl-tRNA synthetase (preKARS2).

## 1. Introduction

Mitochondria are the centres of critical cellular processes, such as oxidative phosphorylation, apoptosis, fatty acids, amino acids and Fe–S cluster metabolisms. Despite this central role, mitochondria present genomes only encoding a fistful of proteins and RNAs dedicated to oxidative phosphorylation or mitochondrial translation. The regulation of mitochondrial activities thus relies on nuclear-encoded factors, which need to be imported in a concerted way in order to tune mitochondrial activity with cytosolic conditions [1,2]. The process of mitochondrial import of cytosolic proteins is far better understood than the process of RNA import, although mitochondrial RNA import has been described in fungi, protozoa, plants and mammals [3].

Regarding tRNAs, the existence of mitochondrial genomes encoding few or no tRNAs in certain organisms such as cniderians or protozoa, respectively, certainly indicates that tRNAs import is necessary for translation [4,5]. However, tRNAs can also be imported in mitochondria of organisms with a full set of mitochondrial DNA-encoded tRNAs. In the yeast *Saccharomyces cerevisiae*, tRNA^Lys^ acceptor 1 (tRK1) is imported despite the presence of a mitochondrial encoded tRNA^Lys^ (tRK3) [6]. In this case, a ~3% fraction of the tRK1 cytosolic pool is constitutively routed towards mitochondria. The import of tRK1 does not confer any obvious advantage to the cell *per se*, except when position 34 of tRK3 becomes hypomodified at non-permissive temperature (37 °C), which creates a dependence of mitochondrial translation upon tRK1 import [7].

Yeast tRK1 can also be imported in human mitochondria *in vitro* [8] and *in vivo* [9], despite that tRNA^Lys^ mitochondrial import has not been demonstrated so far in mammals. At present, the reasons for maintaining this cryptic mechanism in human cells are unknown. The capacity of human cells to import tRK1 into mitochondria points to the possibility to use tRK1 as a vector to target foreign RNA into the mitochondrial matrix. This strategy has been demonstrated experimentally by achieving replacement of the non-functional tRNA^Lys^ in the case of the MERRF syndrome [9], or by the inhibition of the replication of mitochondrial genomic copies harbouring deletions or mutations [10,11,12]. These studies suggest that RNA import mechanisms in yeast and human mitochondria should be related, and the factors involved in tRK1 import in human mitochondria are expected to share the functional characteristics necessary for RNA import in yeast mitochondria [13]. Better understanding of the targeting mechanism in yeast and human cells is critical for optimisation of potential therapeutic approaches.

**Figure 1:**
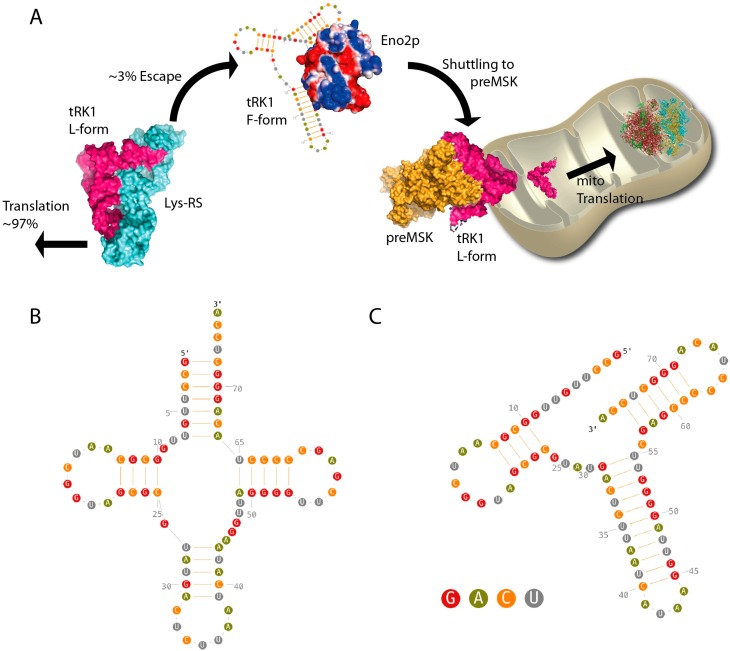
(**A**) Mitochondrial targeting of tRK1 in yeast is achieved by the successive actions of enolase 2 and the precursor of the mitochondrial lysyl-tRNA synthetase (preMSK). At the mitochondrial outer membrane, preMSK takes over enolase to start the import process properly; A fraction of the canonical tRNA L-form (**B**) tRK1 pool is deviated from the cytosolic translation process by the enolase 2, which favors the tRNA conformational change leading to the formation of the F-form (**C**). The D stem-loop is the only domain of the tRNA, which is not scrambled during this process. The nucleotide color code is indicated in panel (**C**).

During the last decade, significant efforts have resulted in identifying the factors responsible for tRK1 mitochondrial targeting in yeast [14,15,16]. According to this mechanism, tRK1 is handled by the glycolytic enzyme enolase in the first place, and further targeted to the mitochondrial membrane where it forms a complex with the precursor of the mitochondrial lysyl-tRNA synthetase (preMSK) (Figure 1A). The RNA determinants, which confer tRK1 import selectivity *versus* tRK2 have been analyzed [17,18] and show that the CUU anticodon and nucleotides from the acceptor arm are critical. These determinants apparently promote the formation of an alternative structure (named the F-form) induced by enolase, composed of three hairpins (Figure 1B,C) [18]. Among those, the D stem-loop is structurally conserved while the two other ones result from reshuffling of the AA- and T-strands of tRK1, which consequently build F form-specific hairpins. Strikingly, from the two enolase isoforms in yeast, only Eno2p allows for tRK1 conformational rearrangement and targeting to preMSK, albeit their sequences are 97% identical.

This mechanism seems to be very close to a potential mechanism in human mitochondria since tRK1 import can be directed *in vitro* using human pre-lysyl-tRNA synthetase (preKARS2) and rabbit enolase [13]. The existence of a cryptic mitochondrial import mechanism of tRNA in human cells and its *in vitro* reconstitution points to human enolases as putative factors in this process.

In the present study, we address the capacity of human enolases to participate in the tRK1 import process by *in vitro* import assay, electrophoretic mobility shift assay (EMSA), and by determining the influence of α, β and γ human enolases on the affinity of preKARS2 for tRK1. Our results show that human enolases promote tRK1 import into mitochondria isolated from HepG2 cells. We also show that enolase is capable of binding to tRK1 and that pre-incubating tRK1 with enolase improves the preKARS2 binding efficiency by decreasing the dissociation constant by one order of magnitude. These results indicate that human enolases participate in the mitochondrial import pathway of tRK1 in human cells together with preKARS2, demonstrating the high similarity between the mechanisms occurring in yeast and human cells.

## 2. Results

**Figure 2:**
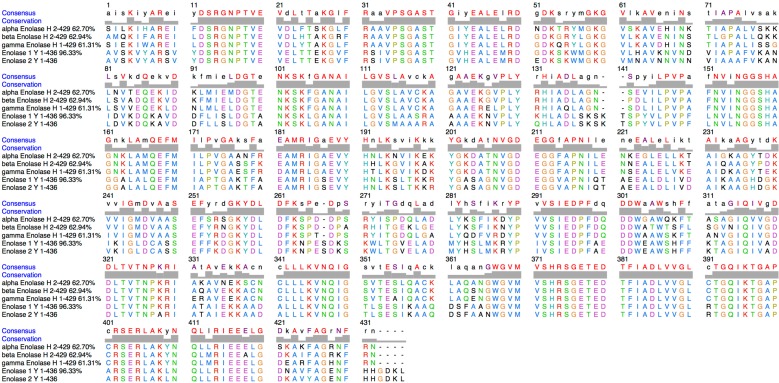
Sequence alignment of the three human enolases and of the two yeast enolases. Genebank Identification of sequences are as follows: α-Enolase 693933; β-Enolase 16878083; γ-Enolase 55669906; Enolase-1 628257676; Enolase 2 6321968.

**Figure 3:**
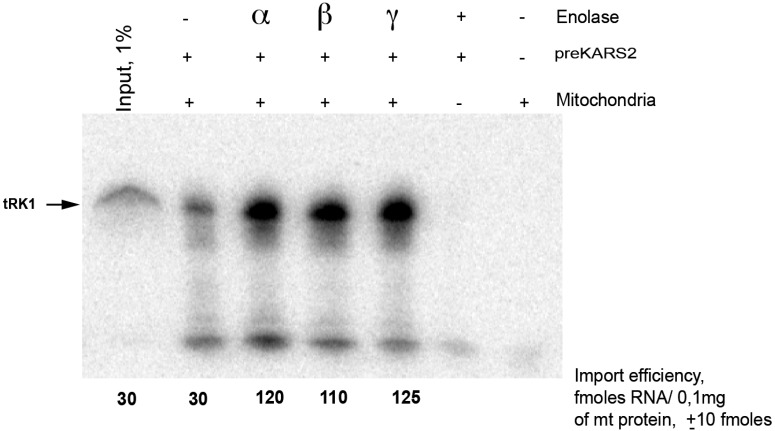
Import of tRK1 into isolated HepG2 mitochondria. An example of the *in vitro* import test, autoradiograph of RNA isolated from purified mitochondria and separated in denaturing 12% polyacrylamide gel (PAAG) is presented. The full-size RNA is indicated by an arrow at the left. Input, 1% of RNA used for each assay (as indicated above the lane), corresponding to 30 fmoles of labeled RNA. Mitochondria (+) correspond to the import assay, Mitochondria (−) to the mock import assay without mitochondria, used as a control for non-specific protein-RNA aggregation. The RNA import efficiency was calculated by comparing the signal with the input and indicated below each lane. One example from three independent experiments is presented, ±SD indicated.

The three human forms of enolase (α, β, γ) are also very conserved and present average identities of 62% with respect to the yeast enolase 2 (Figure 2) [19]. To compare the import-directing capacities of human enolase isoforms, we overexpressed and tag-purified each of the three isozymes of human enolase in *E. coli*. The *in vitro* import test was performed by incubating proteins and labelled RNA with purified mitochondria from HepG2 cells, as described previously [13]. Upon addition of recombinant preKARS2, a small proportion of the tRK1 pool was protected from nuclease degradation (Figure 3), indicating its import into mitochondria. The amount of imported RNA was determined by comparison of the band densities of the protected full-sized RNA isolated from the mitoplasts after the import assay *versus* an aliquot of the input (labelled RNA). tRK1 was very poorly imported with preKARS2 alone. However, its import was significantly increased upon addition of any of the human enolases. Each of the three isozymes demonstrated comparable import-directing capacities (Figure 3). A mock-import test without mitochondria excludes artifactual protection of the RNA by the recombinant proteins. This experiment shows that human counterparts of yeast RNA import factors, the glycolytic enzyme enolase and the cytosolic precursor of mitochondrial lysyl-tRNA synthetase, are sufficient to direct tRK1 import into human mitochondria.

**Figure 4:**
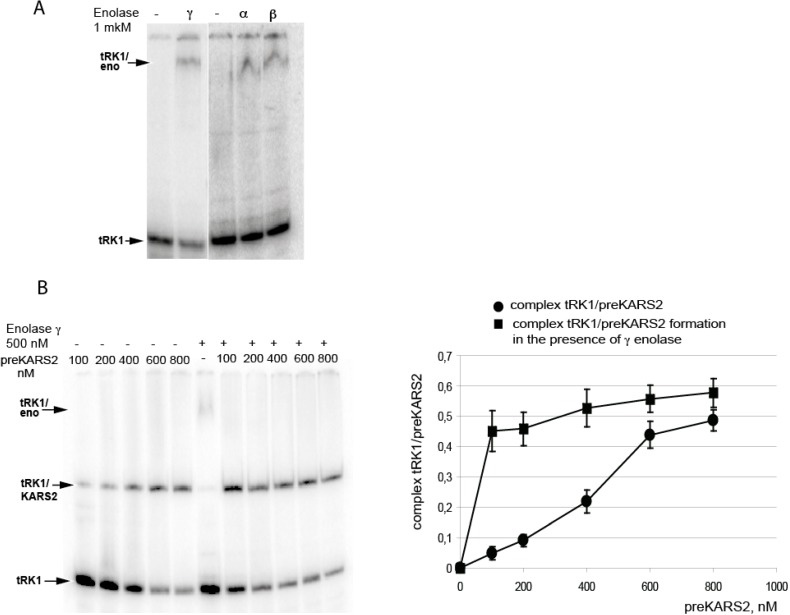
Analysis of RNA-protein interactions by electrophoretic mobility shift assay. (**A**) Autoradiographs of native PAGE-separations of labeled tRK1 in the presence of purified human enolase isoforms; (**B**) The effect of γ enolase on tRK1–KARS2 complex formation. Autoradiograph of the native gel is shown at the **left**, quantification of tRK1–KARS2 complex formation—at the **right**. TRK1, the band corresponding to the free tRK1; RNA-protein complexes are shown with arrows. One example from three independent experiments is presented.

The interaction of purified human enolase isoforms with labeled tRK1 was tested by electrophoretic mobility shift assay (EMSA). We found that all three isoforms of human enolase can form a complex with labelled tRK1 (Figure 4A), with an apparent dissociation constant (*K_d_*) of 2.0 ± 0.5 µM (data not shown). Therefore, human enolase isozymes are capable of interacting with yeast tRNA^Lys^ with the same affinity as yeast enolase Eno2p [15]. When preKARS2 and human γ enolase were present in the mixture, no ternary complex tRK1-preKARS2-enolase was detected. Instead, in the presence of low preKARS2 concentrations, tRK1 was shifted from binding to the enolase to forming a complex with preKARS2 (Figure 4B). Such a pattern is characteristic for consecutive reactions, indicating that the first protein (enolase) binds to the substrate (tRK1) and then transfers it to the second protein (preKARS2). Moreover, the presence of enolase significantly increased the efficiency of tRK1-preKARS2 complex formation (apparent *K_d_* decreased from 300 nM to less than 20 nM).

**Figure 5:**
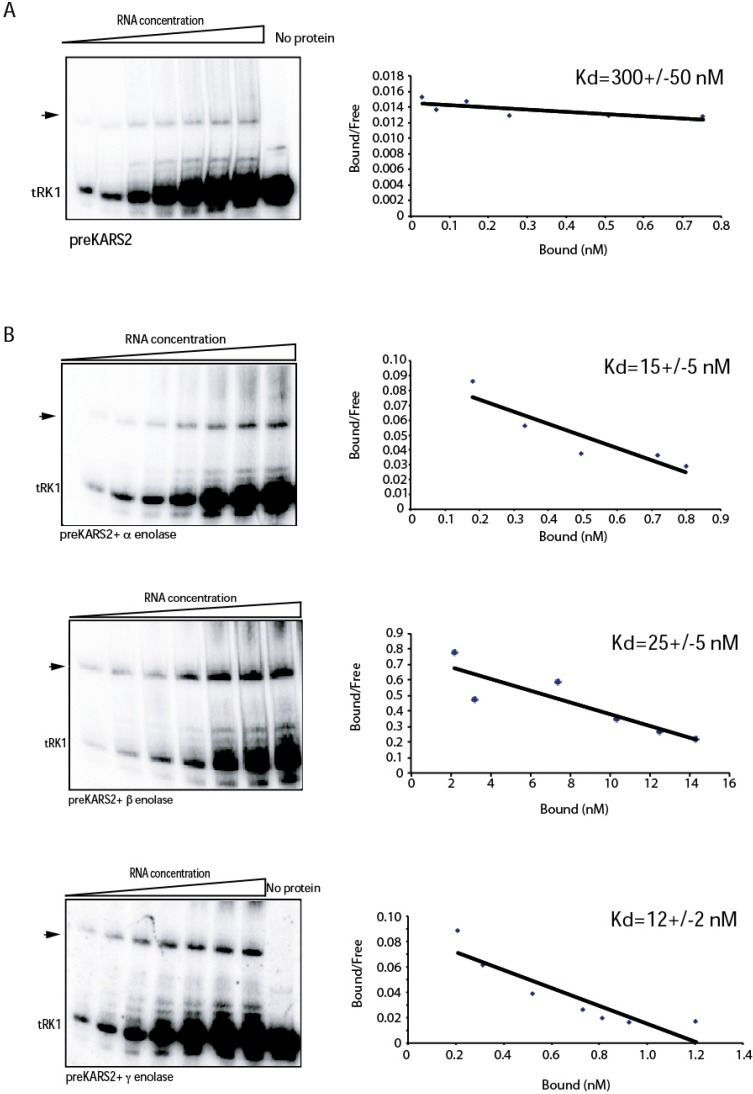
Interaction between labeled tRK1 and preKARS2 in the absence or presence of human enolases. Scatchard plot determination of dissociation constants for preKARS2–tRK1 complex in the absence of enolase (**A**); or in the presence of various isoforms of human enolases, as indicated below the panels (**B**). The bottom band corresponds to the free tRK1; preKARS2–tRK1 complex is shown with an arrow. One example from three independent experiments is presented, *K_d_* ± SD is indicated at the right.

To compare and quantify the effect of the three human enolase isoforms on tRK1-preKARS2 complex formation, we used EMSA followed by Scatchard plot analysis. For this, increasing concentrations of labeled tRK1 (1–50 nM) and fixed concentration of proteins (0.5 µM of preKARS2 and 1 µM of enolase) were used. The results demonstrate that in the absence of enolase, preKARS2 binds tRK1 with a dissociation constant of 300 nM (Figure 4A,B). Addition of enolase isoforms significantly improves the efficiency of tRK1-preKARS2 complex formation resulting in more than 10-fold *K_d_* decrease (from 300 to 12–25 nM) (Figure 5B). These data show that human enolases indeed facilitate the interaction between tRK1 and preKARS2.

## 3. Discussion

*In vitro* assays of tRK1 import in yeast mitochondria have enabled identification of a set of proteins involved in the process. In *Saccharomyces cerevisiae*, enolase 2 (Eno2p) hijacks ~3% of the tRK1 cytosolic pool, which undertakes conformational changes resulting in the formation of a deeply remodeled secondary structure (F-form [18]), which can be transferred to preMSK to be ultimately imported into mitochondria [15]. Strikingly, such a mechanism exists in human cells but remained unsuspected until recently [8,20]. Further investigations have resulted in a better understanding of the tRNA structural requirements [18], as well as in identifying the precursor of human mitochondrial lysyl-tRNA synthetase (preKARS2) as a factor critical for the mitochondrial import of tRNA in human cells and to hypothesize that mammalian enolases could be involved in the process [13]. These studies show a striking similarity between the tRK1 import pathways occurring in yeast and human cells. It is thus reasonable to anticipate that, among the three forms of human enolases, at least one may participate in the import mechanism.

**Figure 6:**
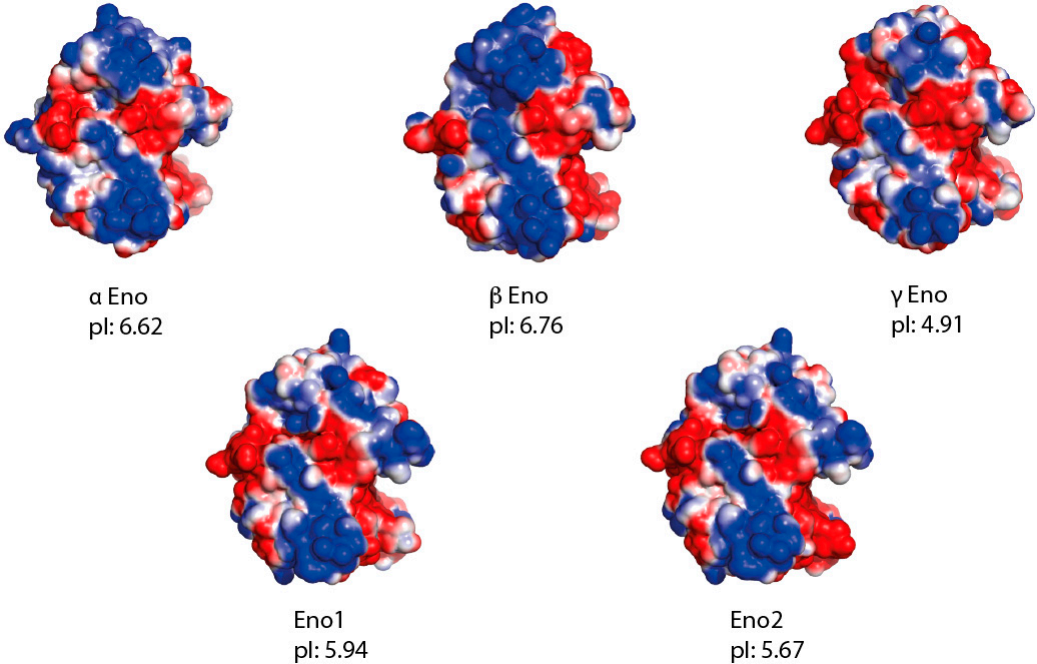
Electrostatic surface potentials visualized from the dimerization interface, corresponding to the region showing the highest density of positively charged residues, which may be responsible for tRK1 binding. Theoretical isoelectric points (pI) are also indicated.

In the present study, we indeed show that the three isoforms α, β and γ of human enolase are able to promote mitochondrial import *in vitro*, interact physically with tRK1 (Figure 3 and Figure 4), and potentialize the affinity of preKARS2 for tRK1 (Figure 5). The human α enolase is expressed ubiquitously, while β and γ are muscle and neuron specific, respectively [21]. The human enolases are very similar to yeast Eno2p with average identities of 62% (Figure 2). Their theoretical isoelectric pH (pI) indicate that all are negatively charged at physiological pH, a situation opposite to RNA binding proteins in general, and raises concern about the ability of this protein family to interact with nucleic acids. In order to identify potential binding sites for tRK1 on members of this protein family, electrostatic potential isosurfaces were calculated. Among the five enolase forms studied, the most acidic pI and electrostatic surface potential are observed in the case of γ enolase, which presents scarce positive charges on the solvant interface, despite its ability to bind tRK1 and to promote *in vitro* mitochondrial targeting, as demonstrated in the present work. Strikingly, the largest positively charged patches are concentrated at the dimerization interface (Figure 6). The interaction of enolase with tRK1 may thus interfere with its dimerization, and, therefore, with enzymatic activity, since only enolase dimers are active during glycolysis [19].

However, it was previously demonstrated that the glycolytic activity of enolase 2 is not correlated to its mitochondrial import activity [15]. Enolase is such an abundant protein that biological processes interfering with its enzymatic activity would be negligible for the cell. This assumption is supported by several observations that human enolase is a multifunctional protein. It is active in plasminogen recognition [22], and a form lacking the first 93 *N*-terminal residues encode a DNA binding protein, MBP, which binds the *c-myc* promoter [23]. In some vertebrates, a processed form of enolase also serves as a structural component of the eye lens (τ crystallin) and retains enzymatic activity, albeit reduced [24].

Therefore, our study strongly suggests that the tissue-specific enolase isoforms may perform the moonlighting function in RNA mitochondrial import in human cells, similarly to yeast Eno2p. The latter seems to act as an RNA chaperone, which redirects part of the tRK1 to the mitochondrial import pathway by favoring the structural rearrangement of tRK1, adding to the list of already known moonlighting functions of this protein. The present data support the idea that human cells possess a cryptic tRNA import mechanism that can be activated in the presence of importable RNAs.

## 4. Experimental Section

### 4.1. Expression and Purification of Recombinant Proteins

Purification of recombinant preKARS2 protein was done as described previously [13]. Plasmids containing human cDNAs encoding enolase isoforms ENO1 (α), ENO2 (γ, neuronal) and ENO3 (β, muscle) were purchased from OriGene (Rockville, MD, USA). Coding regions with a *C*-terminal His-tag were inserted into pET30a expression vector. The resulting full-length tagged proteins were expressed in *Escherichia coli* strain BL21 codon plus (DE3)-RIL cells (Stratagene, Agilent technology, Santa Clara, CA, USA). The transformed cells were grown in LB broth to a cell density A_600_ = 0.6, the protein expression was induced by 0.5 mM Isopropyl β-d-1-thiogalactopyranoside. After incubation for 3 h at 30 °C, cells were harvested, treated with 1 mg/mL of lysozyme on ice for 30 min and sonicated 10 × 20 s in 50 mM sodium phosphate buffer (pH 8.0), 300 mM NaCl and 10 mM imidazole. The clarified lysate was applied to Ni-NTA beads (Qiagen, Hilden, Germany) for 2 h at 4 °C. After washing three times with the same buffer containing 20 mM imidazole, enolases were eluted from the beads with 200 mM imidazole, dialyzed against 50 mM Tris-HCl (pH 8.0), 300 mM NaCl and 40% glycerol and stored at −20 °C. The purity of the proteins was checked by SDS-PAGE with Coomassie blue staining.

### 4.2. Electrophoretic Mobility Shift Assay (EMSA)

TRK1 T7 transcripts were obtained *in vitro* using the Ribomax kit (Promega, Fitchburg, WI, USA). Following transcription, the DNA template was removed by digestion with RQ1 RNase-Free DNase (Promega), and RNA was purified by 8 M urea −12% PAGE. After elution and ethanol precipitation, RNA was dephosphorylated with alkaline phosphatase (Boehringer Mannheim, Mannheim, Germany) and labeled at the 5'-end with γ-^32^P-ATP using T4 polynucleotide kinase (Promega). For the RNA binding assay, labeled RNA was denatured at 100 °C and then allowed to slowly cool down to room temperature. The appropriate amounts of protein and labeled RNA were mixed in 20 µL of a buffer containing 20 mM Tris-HCl pH 7.5, 150 mM NaCl, 10 mM MgCl_2_, 5 mM DTT, 10% glycerol, 0.1 mg/mL BSA and incubated at 30 °C for 15 min. The mixture was fractionated by native 8% PAGE in 0.5× Tris-borate buffer (pH 8.3) and 5% glycerol [25], followed by Typhoon-Trio (GE Healthcare, Fairfield, CT, USA) scanning and quantification as described previously [26]. For Scatchard plot analysis, several standard EMSA reactions were performed in which the concentrations of proteins (0.5 mM for preKARS2 and 1 mM for human enolase isoforms) were kept constant, and the concentration of labeled tRK1 varied from 1 to 100 nM. Calculation of the dissociation constant *K_d_* from the linear regression of experimental data was done as described in [27].

### 4.3. In Vitro Import Assay

The *in vitro* import assay was performed as in [8]. For this, purified HepG2 mitochondria were incubated with ^32^P 5' labeled RNA and purified proteins in import buffer: 0.6 M Sorbitol, 20 mM HEPES-KOH (pH 7), 10 mM KCl, 2.5 mM MgCl_2_, 5 mM DDT and 2 mM ATP. For a standard *in vitro* assay, 3 pmoles of labelled RNA were added to 100 µL of reaction mixture containing 0.1 mg of mitochondria (measured by the amount of mitochondrial protein). This corresponds to 100% RNA input. After incubation for 15 min at 34 °C, 50 µg/mL of RNase A (Sigma Aldrich, Saint Louis, MO, USA) was added and the reaction further incubated for 15 min to digest all the RNA that was not imported. Mitochondria were washed three times with buffer containing 0.6 M sorbitol, 10 mM HEPES–KOH (pH 6.7) and 4 mM EDTA, then resuspended in 100 µL of the same buffer and treated with an equal volume of 0.2% digitonin (Sigma) solution to disrupt the mitochondrial outer membrane, followed by purification of mitoplasts. The mitoplast pellet was resuspended in a solution containing 100 mM CH_3_COONa, 10 mM MgCl_2_, 1% SDS and 0.05% Diethylpyrocarbonate (DEPC) and heated at 100 °C for 1 min. RNA was extracted at 50 °C using water saturated acidic phenol. RNA was precipitated with ethanol and fractionated by 12% PAGE containing 8 M urea, followed by quantification by a Typhoon-Trio scanner using Image Quant-Tools software (GE Healthcare). The amount of imported RNA was determined by comparison of the band density of protected full-sized RNA isolated from the mitoplasts after the import assay with an aliquot (2%–5%) of the RNA input.

### 4.4. Protein Alignment, Molecular Modelling and Electrostatic Surface Calculation

Sequences were obtained using blastp [28], aligned by clustalw [29] and visualized using chimera [30]. The three-dimensional molecular model of Eno2p was obtained by automatic homology modeling using the SWISS-MODEL pipeline [31]. The best template was 2al1 [32] from the protein databank. Electrostatic potential surfaces were represented in PyMOL [33] using PDB2PAQR [34] and APBS [35] plugins. Theoretical pI were determined using ExPASy tools [36].
